# The BAF chromatin remodelling complex is an epigenetic regulator of lineage specification in the early mouse embryo

**DOI:** 10.1242/dev.131961

**Published:** 2016-04-15

**Authors:** Maryna Panamarova, Andy Cox, Krzysztof B. Wicher, Richard Butler, Natalia Bulgakova, Shin Jeon, Barry Rosen, Rho H. Seong, William Skarnes, Gerald Crabtree, Magdalena Zernicka-Goetz

**Affiliations:** 1 Wellcome Trust Cancer Research UK Gurdon Institute, Tennis Court Road, Cambridge CB2 1QN, UK; 2 Department of Physiology, Development and Neuroscience, University of Cambridge, Cambridge CB2 3DY, UK; 3 Bateson Centre andDepartment of Biomedical Science, University of Sheffield, Western Bank, Sheffield S10 2TN, UK; 4 Department of Biological Sciences, Institute of Molecular Biology and Genetics, Seoul National University, Seoul 151-747, South Korea; 5 Wellcome Trust Sanger Institute, Hinxton CB10 1SA, UK; 6 Department of Developmental Biology, Stanford University Medical School, Stanford, CA 94305, USA

**Keywords:** BAF complex, Chromatin remodelling, Epigenetics, Lineage specification, Mouse embryo, Pluripotency, SMARCC1

## Abstract

Dynamic control of gene expression is essential for the development of a totipotent zygote into an embryo with defined cell lineages. The accessibility of genes responsible for cell specification to transcriptional machinery is dependent on chromatin remodelling complexes such as the SWI\SNF (BAF) complex. However, the role of the BAF complex in early mouse development has remained unclear. Here, we demonstrate that BAF155, a major BAF complex subunit, regulates the assembly of the BAF complex *in vivo* and regulates lineage specification of the mouse blastocyst. We find that associations of BAF155 with other BAF complex subunits become enriched in extra-embryonic lineages just prior to implantation. This enrichment is attributed to decreased mobility of BAF155 in extra-embryonic compared with embryonic lineages. Downregulation of BAF155 leads to increased expression of the pluripotency marker *Nanog* and its ectopic expression in extra-embryonic lineages, whereas upregulation of BAF155 leads to the upregulation of differentiation markers. Finally, we show that the arginine methyltransferase CARM1 methylates BAF155, which differentially influences assembly of the BAF complex between the lineages and the expression of pluripotency markers. Together, our results indicate a novel role of BAF-dependent chromatin remodelling in mouse development via regulation of lineage specification.

## Abstract (precis)

**Summary:** Associations of BAF155 with other BAF complex subunits are enriched in extra-embryonic lineages prior to implantation, while changes in BAF155 levels modulate the expression of early developmental markers.

## INTRODUCTION

Differentiation involves a cascade of cell fate decisions that progressively limit the potential of a cell to contribute to other lineages. Two cell fate decisions take place in the pre-implantation mouse embryo. The first of these causes the separation of the inner cell mass (ICM) from trophectoderm (TE), the first extra-embryonic lineage; the second leads to the formation of two distinct cell populations from the ICM: pluripotent epiblast (EPI) and primitive endoderm (PE), which forms the second extra-embryonic lineage (Schrode et al., 2013; Zernicka-Goetz et al., 2009). The identity of cells contributing to each of these lineages is maintained by a regulatory network, which is governed by master gene regulators (Nichols and Smith, 2009). *Nanog*, *Oct4* (*Pou5f1*) and *Sox2* are central to the gene network that maintains the pluripotent state of the EPI, *Cdx2* and *Eomes* are required for TE differentiation, and *Gata4*, *Gata6* and *Sox17* are required to direct PE specification. Differential behaviour of the protein complexes and chromatin-modifying enzymes that alter the structure of chromatin is required in concert with transcription factors to regulate appropriate gene expression for these processes (Paul and Knott, 2014).

One of the first described epigenetic regulators involved in lineage specification in the mouse embryo is the arginine methyltransferase CARM1 (Torres-Padilla et al., 2007). Elevated levels of CARM1 lead to increased expression of NANOG and SOX2 and the preferential contribution of blastomeres to the ICM (Torres-Padilla et al., 2007; Parfitt and Zernicka-Goetz, 2010). The effect of CARM1 could be attributed to modification of specific arginine residues on histone H3, which skews the differentiation potential of the blastomere towards pluripotency. However, it also remains possible that methylation by CARM1 contributes to chromatin remodelling, as shown recently in other model systems (Wang et al., 2014).

The SWI\SNF (BAF) complex plays important roles in the proliferation and differentiation of various cell types (Ho and Crabtree, 2010). Chromatin remodelling by the BAF complex was thought to be an exclusively permissive mechanism necessary for gene transcription. However, the BAF complex was found to have an instructive role in gene expression in certain cell types through its combinatorial assembly and interactions with tissue-specific transcription factors (Lessard et al., 2007; Nie et al., 2000; Wu et al., 2007). For instance, in embryonic stem cells (ESCs) the BAF complex occupies the promoters of nearly all of the genes in the core pluripotency network and directly interacts with OCT4 and SOX2 to refine the transcription of genes involved in pluripotency and self-renewal (Ho et al., 2009a,b, 2011).

BAF complexes are polymorphic assemblies of up to 15 subunits encoded by 29 genes (Kadoch et al., 2013). The biological specificity of the complexes is thought to emerge from combinatorial assembly of the products of the families of genes that encode the different subunits. Subunits of the complexes have been implicated in various processes, such as tumour suppression and development of the nervous system (Kadoch et al., 2013; Kadoch and Crabtree, 2013). Null mutations in genes encoding several of the BAF complex subunits, such as BRG1 (SMARCA4), BAF155 (SMARCC1) and BAF47 (SMARCB1), lead to developmental arrest at the pre- to post-implantation transition (Bultman et al., 2000; Guidi et al., 2001; Kim et al., 2001; Klochendler-Yeivin et al., 2000). The primary reason for developmental arrests at this embryonic stage has not been determined to date. In order to establish the underlying basis for these developmental defects, here we examine the role of BAF155, a major component of the BAF complex, in late pre-implantation stages of mouse embryos.

## RESULTS

### Expression and proximity of BAF complex subunits in embryonic and extra-embryonic lineages

**Fig. 1.**
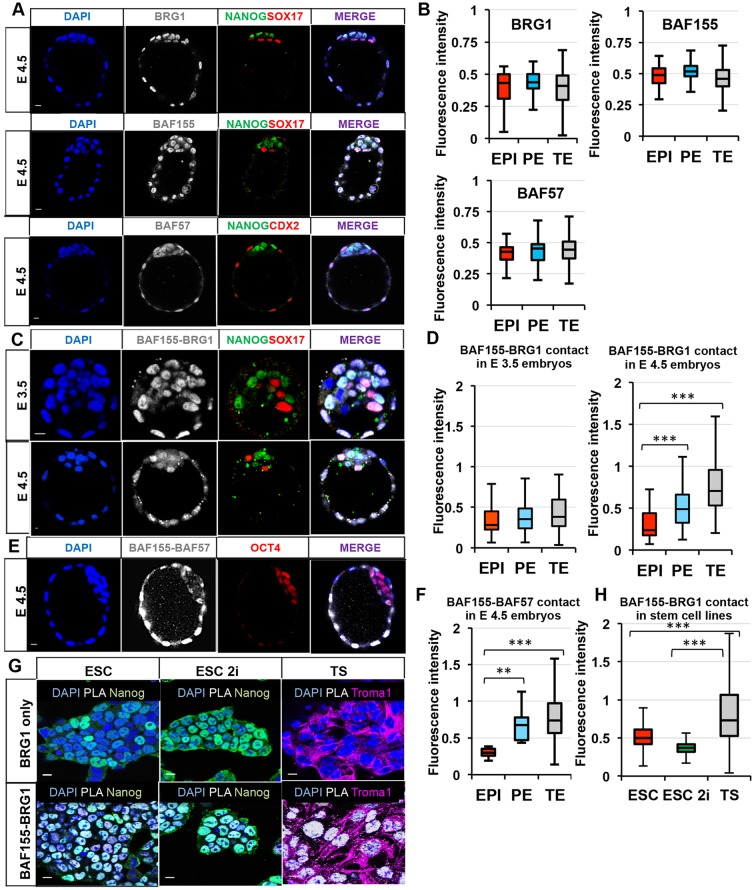
**Associations between the BAF complex subunits are upregulated in extra-embryonic lineages at the late blastocyst stage.** (A) Antibody staining of BAF complex subunits (*n*≥9 each). (B) Automated quantification of fluorescence intensity of *z*-stacks in the three distinct cell types of the blastocyst. (C) Fluorescent signal generated by PLA shows the interaction between BAF155 and BRG1 in early and late blastocysts. (D) The fluorescence intensity of BAF155-BRG1 contact is similar among the lineages at E3.5, but higher in TE and PE at E4.5. (E) The fluorescence intensity from BAF155-BAF57 interaction is increased in TE and PE. (F) Quantification of fluorescent signal from BAF155-BAF57 PLA between the embryonic lineages. (G) Fluorescent signal generated by BAF155-BRG1 PLA in stem cell lines. Troma1 antibody detects the TE marker cytokeratin 8. (H) The fluorescence intensity of BAF155-BRG1 contact is higher in TSC (median, 0.73; mean, 0.88) versus ESC (median, 0.50; mean, 0.52) and ESC 2i (median and mean, 0.37). Error bars represent s.d. **P<0.01, ***P<0.001, ANOVA. Scale bars: 10 µm.

The key process that has to be established in the mammalian embryo by implantation is the specification of three distinct lineages: pluripotent EPI and the differentiated extra-embryonic lineages PE and TE. To investigate whether the chromatin remodelling mediated by the BAF complex participates in this process, we first examined its localisation when all three lineages are established at late blastocyst stage (E4.5). We analysed the distribution of BAF complex subunits: a catalytic subunit BRG1; a scaffolding subunit BAF155 and its homologue BAF170 (SMARCC2); and subunit BAF57, which contributes to DNA binding (Link et al., 2005; Chi et al., 2002; Phelan et al., 1999; Sohn et al., 2007). We found that BRG1, BAF155 and BAF57 are expressed in all cell lineages, whereas BAF170 is not expressed (Fig. 1A, Fig. S1A). To confirm these observations, we quantified the expression levels of BRG1, BAF155 and BAF57 between the lineages using NANOG, SOX17 or CDX2 as markers of EPI, PE and TE lineages, respectively. The automated quantification of BRG1, BAF155 and BAF57 revealed no significant differences in signal between the three cell types (*P*>0.05, ANOVA), indicating that these subunits are expressed equally in all the lineages at E4.5 (Fig. 1B).

As the association of BRG1 and BAF155 is a distinctive feature of the BAF complex in ESCs (Ho et al., 2009b), we next sought to determine whether these subunits are in close proximity and therefore have the potential to act as a functional complex in the embryo. We used the proximity ligation assay (PLA), which results in a fluorescent signal when proteins neighbour each other (Soderberg et al., 2006). We found that at the early blastocyst stage (E3.5), BAF155 and BRG1 are in close proximity throughout the embryo regardless of cell lineage (Fig. 1C, *n*=16). However, at the late blastocyst stage (E4.5) there was a substantial increase in BAF155-BRG1 proximity in the TE and PE in comparison to EPI (Fig. 1C, *n*=18). To confirm these results, we quantified the fluorescence intensity representing BAF155 and BRG1 proximity in E3.5 and E4.5 blastocysts as detected by PLA (Fig. 1D). There was no difference in the fluorescent signal reflecting BAF155-BRG1 proximity between the three lineages at E3.5 (*P*>0.05, ANOVA), whereas the signal was increased 2.6-fold in the TE at E4.5 in comparison to EPI (*P*<0.001, ANOVA), and to a lesser extent (1.5-fold) in the PE (*P*<0.001, ANOVA). A similar pattern was detected at E4.5 for BAF155 and BAF57 (Fig. 1E,F, *n*=6). A signal could not be detected when one of the antibodies was omitted (Fig. S1B, *n*=12).

To determine whether the PLA signal was specific, we carried out several control experiments. We first knocked down BAF155 by dsRNA (see also Fig. 3) and found that the PLA signal between BAF155 and BRG1 was strongly reduced (Fig. S1C, *n*=11). We next assessed the proximity of BRG1 to CENPA, a subunit of the centromere complex suggested not to directly interact with BRG1 (data obtained from the interactome library http://string-db.org), and could not detect a PLA signal (Fig. S1D,D′, *n*=20). To examine whether the differences in proximity of BAF complex components might be due to differences in the physical accessibility of cells to the PLA procedure, we examined the relationship of histone H2A and the histone modification H3K9me3. Consistent with previous work (Wongtawan et al., 2011; Nashun et al., 2010), histone H2A and H3K9me3 were distributed equally in the three lineages when assessed by immunostaining (Fig. S1E,E′) and PLA (Fig. S1E″), suggesting that the physical properties of cells in different lineages of a blastocyst are unlikely to influence the outcome of the PLA.

We also analysed the proximity of BAF155 and BRG1 in cell lines derived from the respective lineages: ESCs cultured in LIF, representative of ICM; ESCs cultured in 2i medium and LIF, representative of EPI (Boroviak et al., 2014); and trophoblast stem cells (TSCs), derived from TE. This revealed significantly increased proximity of these subunits in TSCs compared with the other cell lines (Fig. 1G,H, *P*<0.001, ANOVA). Together, these results indicate that, at the late blastocyst stage, the association of the BAF complex subunit BAF155 with BRG1 and BAF57 is increased in the extra-embryonic lineages in comparison to the EPI.

### BAF155 regulates BAF complex subunit associations in a mouse embryo

**Fig. 2.**
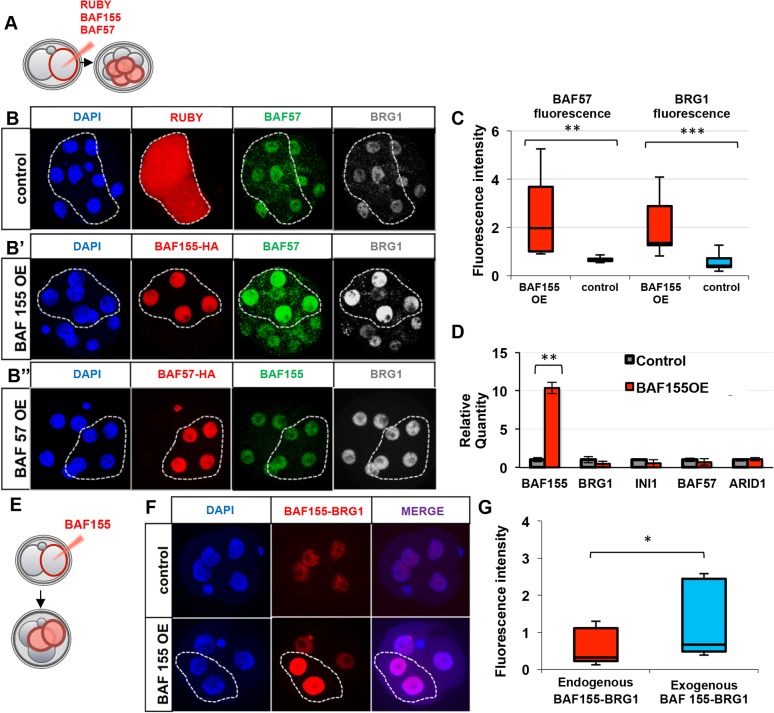
**Upregulation of BAF155 causes upregulation of BAF complex components.** (A) HA-tagged human *BAF155*, mouse *Baf57* or *Ruby* mRNAs were injected into one blastomere at the 2-cell stage and analysed at the 8-cell stage. (B-B″) Clonal overexpression of BAF155 results in the upregulation of protein levels of the complex subunits (B′), whereas overexpression of Ruby (B) or BAF57 (B″) does not. (C) The protein levels of BAF57 and BRG1 upon BAF155 overexpression (OE) were upregulated by ∼3-fold. (D) qRT-PCR analysis of transcripts for key components of the BAF complex 24 h after BAF155 OE. INI1 refers to *Baf47* (*Smarcb1*). (E) HA-tagged *BAF155* mRNA was injected into one blastomere at the 2-cell stage and analysed at the 4-cell stage by PLA. (F) Clonal BAF155 OE caused an increase in BAF155-BRG1 interaction in the injected clones (dashed outline). (G) Overexpression of exogenous BAF155 resulted in 2-fold upregulation of BAF155-BRG1 contact. Error bars represent s.d. *P<0.05, **P<0.01, ***P<0.001, Student's *t*-test.

Since BAF155 has the unique ability to positively regulate the levels of major components of the BAF complex *in vitro* (Sohn et al., 2007), we next determined whether upregulation of BAF155 would have the same effect *in vivo*. We injected human *BAF155* mRNA tagged with hemagglutinin (HA) into one blastomere at the late 2-cell stage and analysed embryos at the 8-cell stage. This produced embryos in which half of the cells overexpressed BAF155 and the other half had an endogenous level of BAF155, therefore serving as a control (Fig. 2A). Increasing the BAF155 protein level 2.3-fold resulted in a ∼3-fold increase in the levels of BAF57 and BRG1 protein (Fig. 2B′,C, *n*=16; Fig. S2A,B). Overexpression of the fluorescent protein mRuby (*n*=23) or BAF57 (*n*=13), as controls, to a comparable protein level did not lead to a similar increase (Fig. 2B,B″, Fig. S2A-D), indicating a specific effect of BAF155. To address whether the increased BAF155 expression led to changes in transcript levels of BAF subunits, we performed qRT-PCR 24 h after *BAF155* mRNA injection (Fig. 2D). There were no significant changes in the levels of transcripts encoding key components of the BAF complex (*P*>0.05, Student's *t*-test), suggesting that the increase in BRG1 and BAF57 upon BAF155 upregulation occurs at the post-transcriptional level.

Since the observed correlation between the level of BAF155 and other complex subunits suggested that new BAF chromatin remodelling complexes might be formed following overexpression of BAF155, we determined whether exogenous BAF155 could associate with the endogenous complex. We injected HA-tagged *BAF155* mRNA into one blastomere at the late 2-cell stage and analysed the extent of proximity between exogenous BAF155-HA and endogenous BRG1 using PLA at the 4-cell stage (Fig. S2E-G, *n*=12). The specificity of incorporation was confirmed technically by omitting one of the antibodies (Fig. S2G′, *n*=7), and biologically since no interaction was detected between HA-tagged CENPA and BRG1 (Fig. S2G″, *n*=13). The PLA detected a 2-fold increase in proximity between total BAF155 and endogenous BRG1 in the blastomeres expressing exogenous BAF155 (Fig. 2E-G, *n*=11, *P*<0.05, Student's *t*-test). These findings indicate that the level of BAF155 can modulate BAF complex subunit associations.

### The level of BAF155 affects the expression of lineage markers in the mouse blastocyst

**Fig. 3.**
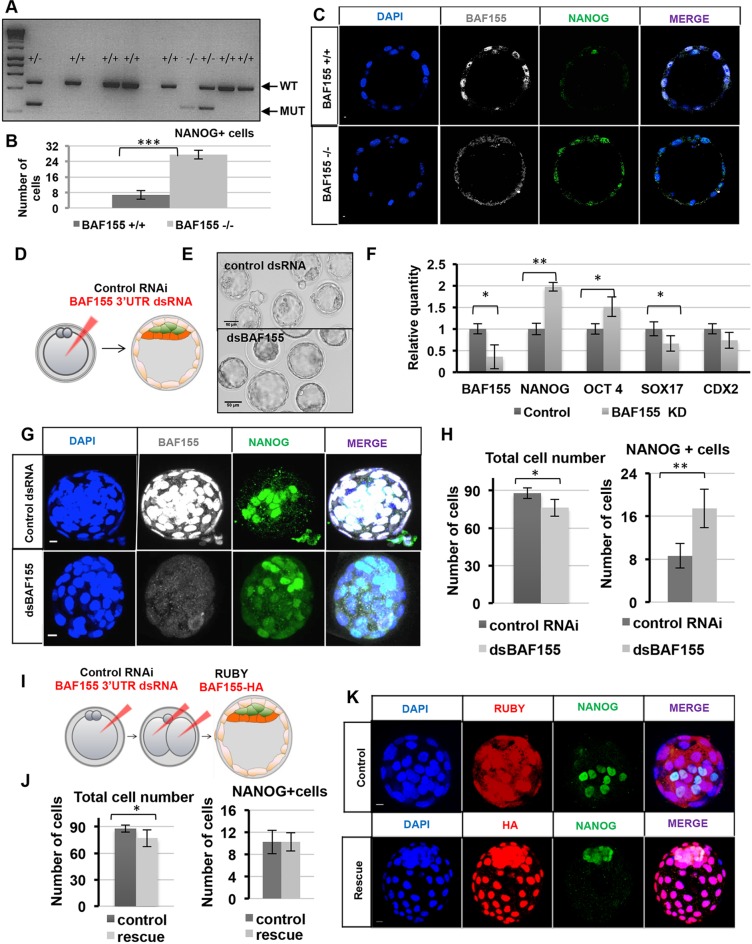
**BAF155 is required for *Nanog* downregulation during lineage specification at the blastocyst stage.** (A) Three-primer single-embryo PCR analysis from *Baf155* heterozygous intercrosses showing wild-type (450 bp) and mutated (250 bp) alleles. The first lane contains a size marker (1 kb HyperLadder). (B) *Baf155*^−/−^ embryos have a significantly increased number of NANOG^+^ cells compared with control littermate embryos (***P<0.01, Student's *t*-test). (C) *Baf155*^−/−^ embryos exhibit ectopic nuclear expression of NANOG in TE (determined morphologically), unlike *Baf155*^+/+^ littermates that only have nuclear NANOG expression in the EPI cells. (D) Downregulation of BAF155 at the zygote stage was performed using dsRNA against the 3′UTR (dsBAF155), or control dsRNA. (E) DIC images of embryos injected with control dsRNA or dsBAF155. (F) qRT-PCR of whole embryos, comparing lineage marker transcripts of control and dsBAF155 blastocysts. (G) *z*-projections of immunofluorescent images of control and dsBAF155 E4.5 blastocysts. (H) The total number of cells in control E4.5 blastocysts (88±4) was slightly reduced compared with dsBAF155 blastocysts (76±7). The number of NANOG^+^ cells in dsBAF155 blastocysts was increased (17±4) compared with the control (9±3). (I) Rescue experiment of BAF155-depleted embryos. (J) Rescue blastocysts had fewer cells (77±9) than control blastocysts (87±5), but the same number of NANOG^+^ cells. (K) No ectopic expression of NANOG in TE was detected in the majority of rescued blastocysts. Error bars represent s.d. *P<0.05, **P<0.01, ***P<0.001, Student's *t*-test. Scale bars: 10 µm.

Null mutations of several BAF complex subunits lead to developmental arrest at the pre- to post-implantation transition (Bultman et al., 2000; Guidi et al., 2001; Kim et al., 2001; Klochendler-Yeivin et al., 2000). To understand the cause of this developmental arrest, we next examined the consequences of depleting the levels of BAF155. We first examined *Baf155* knockout embryos just prior to implantation at E4.5, generated by the intercross between heterozygous *Baf155* knockout parents (Kim et al., 2001), the genetic identity of which we confirmed by single-embryo genotyping (Fig. 3A). We found no obvious morphological defects at this embryonic stage; however, after careful examination we found that 18% of embryos from such intercrosses displayed ectopic expression of NANOG in TE in comparison to control wild-type littermates (Fig. 3B,C, *n*=22). To investigate this phenotype in more detail, we next examined the embryos after BAF155 downregulation using dsRNA against the *Baf155* 3′UTR injected into the zygote (Fig. 3D). Similarly to *Baf155* knockout embryos, BAF155 protein was poorly detectable by immunofluorescence in RNAi embryos at E4.5 and the *Baf155* mRNA level was reduced by more than 65% (Fig. 3F,G, *n*=24). In agreement with the phenotypic effect of *Baf155* knockout (Kim et al., 2001), BAF155 downregulation did not lead to any obvious morphological defects until the time of blastocyst hatching from the zona pellucida, just before implantation (Fig. 3E). Examination of BAF155 knockdown embryos revealed significantly increased expression of the pluripotency marker *Nanog* (on both mRNA and protein levels) and ectopic expression of NANOG in the TE lineage (Fig. 3F-H, *n*=24). The transcript levels of another pluripotency marker, *Oct4*, were also upregulated, whereas *Sox17* levels were decreased, although we did not observe misexpression of SOX17 and CDX2 by immunofluorescent staining (Fig. 3F, Fig. S3A,B, *n*=24). The total cell number was slightly, but significantly, decreased upon BAF155 downregulation (Fig. 3H, *P*<0.05, Student's *t*-test), indicating a developmental defect.

To determine whether the consequences of BAF155 downregulation by RNAi are specific, we tested whether restoring the BAF155 level could prevent the ectopic expression of NANOG by injecting HA-tagged *BAF155* mRNA into both blastomeres of embryos zygotically depleted of endogenous BAF155 (Fig. 3I, *n*=14). We found that by E4.5 the ectopic expression of NANOG in TE cells was undetectable in 85.7% of embryos (Fig. 3J,K, *n*=14) suggesting specificity of the *BAF155* dsRNA depletion. To validate the dsRNA knockdown phenotype further, we injected siRNA against the coding sequence of *Baf155* mRNA into the zygote. BAF155 siRNA (*n*=16) led to a reduction in *Baf155* mRNA and protein compared with control siRNA embryos (*n*=12) (Fig. S3C-E). Similarly to *Baf155* knockout and *BAF155* dsRNA, depletion with siRNA resulted in an increase in the number of NANOG-expressing cells (Fig. S3D) and upregulation of *Nanog* mRNA levels, as well as decreased expression of *Sox17* (Fig. S3C). Taken together, these results indicate that depletion of BAF155 leads to increased expression of *Nanog* in the ICM and its ectopic expression in TE.

**Fig. 4.**
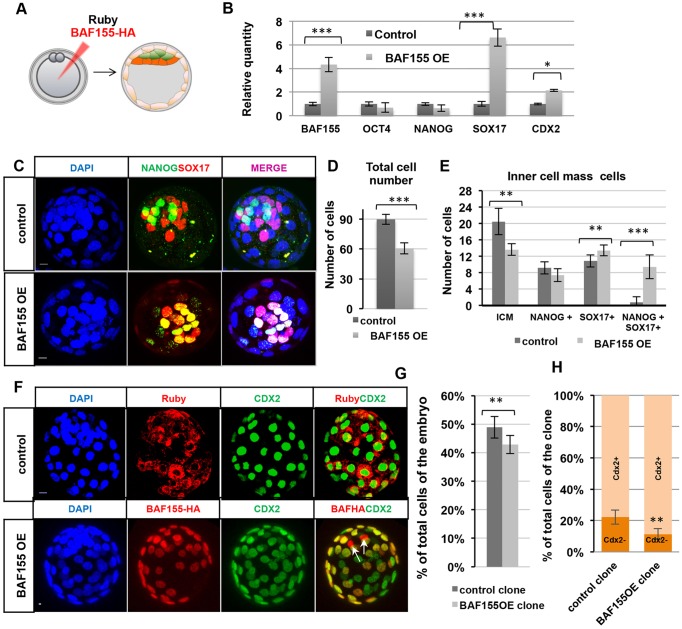
**Upregulation of BAF155 shifts the developmental programme towards the extra-embryonic lineage.** (A) Overexpression (OE) experiments of BAF155 using the HA-tagged BAF155 construct or of Ruby (control). (B) qRT-PCR on whole embryos, comparing lineage marker transcripts of control and BAF155 OE blastocysts. (C) Immunofluorescence images of control and BAF155 OE blastocysts at E4.5. (D) Total cell number was reduced in BAF155 OE blastocysts (61±6) compared with the control (90±5). (E) The total number of ICM cells was reduced in BAF155 OE blastocysts (14±2) compared with the control (21±3); the majority of ICM cells in BAF155 OE blastocysts co-express NANOG and SOX17 (9±3), unlike control blastocysts (1±2). (F) *z*-projections of control and BAF155 OE blastocysts: Ruby blastocyst contributes equally to the ICM and CDX2^+^ TE cell populations, whereas BAF155 OE blastocyst infrequently contributes to the CDX2^−^ ICM cells (arrows). (G) The percentage of clones injected with BAF155 contributing to the total blastocyst was lower than that injected with Ruby. (H) Clones injected with BAF155 showed a higher contribution to the CDX2^+^ TE lineage compared with Ruby^+^ clones. Error bars represent s.d. *P<0.05, **P<0.01, ***P<0.001, Student's *t*-test. Scale bars: 10 µm.

To determine whether upregulation of BAF155 would phenotypically have a counter effect, we injected HA-tagged *BAF155* mRNA into zygotes (Fig. 4A-E). Although embryos developed until early blastocyst stage, their development arrested at the E3.5 to E4.5 transition, with a significantly decreased cell number compared with controls (*n*=9, *P*<0.001, Student's *t*-test; Fig. 4C,D, Fig. S4, Movies 1 and 2). qRT-PCR analysis revealed that a 5-fold increase of *Baf155* mRNA at E4.5 was associated with a 7-fold increase in *Sox17* mRNA and a 2-fold increase in *Cdx2* mRNA (Fig. 4B, *n*=30, *P*<0.01, Student's *t*-test). Additionally, unlike in control blastocysts in which ICM cells (21 on average, *n*=6 embryos) fell into distinct NANOG^+^ (EPI) and SOX17^+^ (PE) populations and only very rarely did ICM cells show co-expression of these markers (one or two cells, on average), in BAF155-overexpressing E4.5 blastocysts the majority of ICM cells (14 cells, *n*=9 embryos) showed co-expression of SOX17 and NANOG (9 cells on average, *P*<0.01, Student's *t*-test, Fig. 4C,E) suggesting defects in EPI versus PE lineage specification.

The finding that BAF155 upregulation resulted in lineage specification defects prompted us to ask whether BAF155 upregulation in one blastomere at the 2-cell stage would also affect lineage contribution (Fig. 4F-H). We found that the clones overexpressing BAF155 were reduced in number and contributed preferentially to the extra-embryonic lineage (CDX2^+^ cells) rather than to the ICM (CDX2^−^ cells) (Fig. 4G,H, *n*=12). Specifically, only 11.4% of the BAF155-overexpressing clones contributed to the CDX2^−^ population compared with 22.2% of clones expressing the control mRNA *Ruby* (*n*=11). Therefore, BAF155 upregulation in a single 2-cell blastomere biases its progeny towards the extra-embryonic lineage (Fig. 4H, *P*<0.01, Student's *t*-test).

Together, our results indicate that the level of BAF155 is important for accurate lineage specification by implantation: decreased levels of BAF155 lead to increased expression of the pluripotency genes *Nanog* and *Oct4* and, counter to this, increased BAF155 results in upregulated expression of the differentiation genes *Cdx2* and *Sox17*.

### BAF155 differentially affects the dynamics of BAF component assembly between embryonic and extra-embryonic lineages

**Fig. 5.**
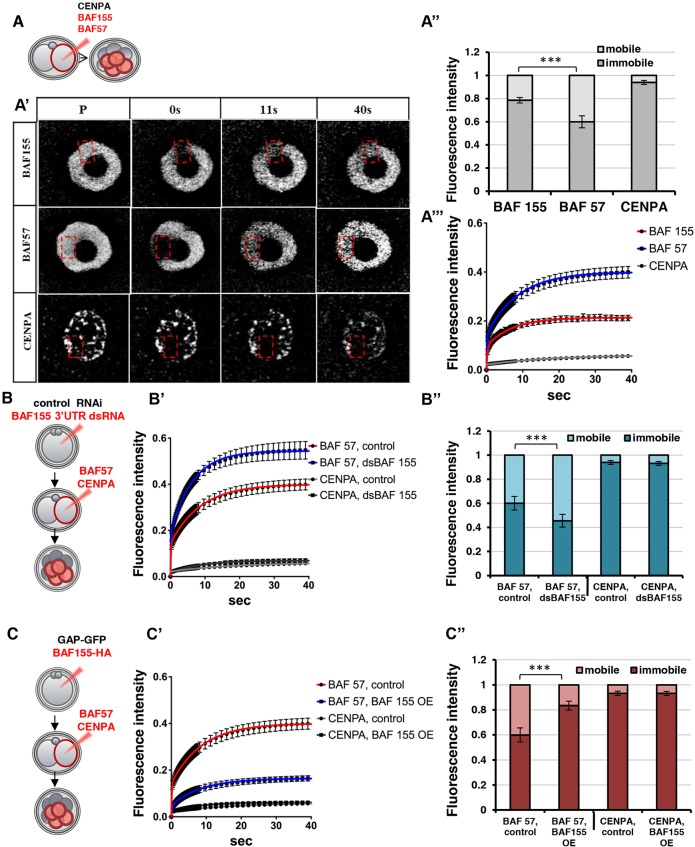
**The mobility of BAF57 is dependent on the level of BAF155 expression.** (A) The live kinetics of BAF155, BAF57 and CENPA proteins tagged with mCherry were measured at the 8-cell stage. (A′) Recovery kinetics were estimated by measuring fluorescence intensity (within the boxed region) prior to photobleaching (P) and during 40 s after photobleaching. (A″) The immobile pool was significantly greater for BAF155 (78.61±2.4%) than BAF57 (59.97±5.0%); the immobile fraction of CENPA was the highest (93.9±1.7%). (A‴) Greater mobility of BAF57 than of BAF155 or CENPA was detected by greater FRAP recovery. (B) The kinetics of BAF57 and CENPA measured at the 8-cell stage of embryos zygotically depleted with dsBAF155. (B′) Recovery of BAF57 was increased in dsBAF155 embryos compared with the controls, whereas CENPA mobility was unaffected. (B″) The immobile fraction of BAF57 in dsBAF155 embryos was reduced, but remained unaffected for CENPA. (C) Kinetics of BAF57 and CENPA estimated at the 8-cell stage of embryos injected with BAF155-HA. (C′) The recovery of BAF57 was reduced in BAF155 OE embryos compared with the control, whereas in CENPA embryos the rate was unchanged. (C″) The immobile fraction of BAF57 was significantly increased in BAF155 OE (83.4±3.6%) compared with control (60±5.6%), whereas CENPA was unaffected. Error bars represent s.e.m. ***P<0.001, *F*-test.

Since the above results suggested that the BAF complex might, to a certain extent, be dynamic, we investigated this possibility by an independent method. We examined the mobility of two BAF complex subunits, BAF155 and BAF57, using fluorescence recovery after photobleaching (FRAP). BAF155 and BAF57 were labelled with the fluorescent tag mCherry, which does not interfere with the association of these subunits with BRG1, as shown by PLA (Fig. S5A). To provide a control in the form of a stable nuclear protein complex, we similarly tagged CENPA, which is known to be stably associated with centromeres (Hemmerich et al., 2008; Hellwig et al., 2008). The constructs were injected into one blastomere of 2-cell embryos, while the uninjected blastomere provided an internal control, and FRAP was carried out on a defined area of the nucleus at the 8-cell stage (Fig. 5A,A′). Measurements of the maximum fluorescence recovery were used to assess the mobility of these proteins in terms of the ratio of bound (immobile) versus unbound (mobile) protein.

A stable complex typically shows a large immobile pool of protein subunits with very little unbound protein (Hemmerich et al., 2011; Carrero et al., 2003). As expected, CENPA, a protein that remains immobile on chromatin throughout most of the cell cycle, showed a recovery FRAP curve indicating that 94% (±1.7%) of the total protein is immobile (*n*=22), in agreement with a previous report (Hemmerich et al., 2008). By contrast, FRAP curves for the two components of the BAF complex showed different kinetics. Both fitted bi-exponential curves, but with 78±2.4% (*n*=18) of BAF155 compared with 59.9±5.0% (*n*=20) of BAF57 being immobile under the same conditions (*P*<0.001, *F*-test, Fig. 5A′,A″). These results suggest that although the majority of the BAF155 and BAF57 subunits exist in a stable complex, there is a pool of each protein in the embryo that is mobile.

To determine whether the immobile fraction of BAF57 would change upon BAF155 downregulation, we depleted BAF155 at the zygote stage (Fig. S5B), and investigated the mobility of BAF57 and CENPA by FRAP at the 8-cell stage (Fig. 5B, Fig. S5D,E). The immobile fraction of CENPA (*n*=10) remained unaffected (93.1±1.7% compared with 94±1.6% in controls, *P*>0.05, *F*-test; Fig. 5B′,B″). By contrast, the immobile fraction of BAF57 was decreased upon BAF155 downregulation (45±5.2% compared with the control 60±5.6%, *P*<0.001, *F*-test; Fig. 5B′,B″), suggesting that the level of BAF155 can affect the proportion of BAF57 subunit associated within the complex. As BAF155-depleted cells tend to upregulate *Nanog* expression (see above), a decrease in the immobile fraction of BAF complex components would suggest that expression of the pluripotent gene *Nanog* is associated with a reduction in stable BAF complex.

To determine whether upregulation of BAF155 might have a reciprocal effect, we overexpressed BAF155-HA at the zygote stage (Fig. S5C) and used FRAP to investigate the kinetics of BAF57 and CENPA proteins at the 8-cell stage (Fig. 5C, Fig. S5F,G). This resulted in an increase in the immobile fraction of BAF57 to 83.4±3.6% (*n*=11) compared with 60±5.6% in embryos with endogenous BAF155 levels (Fig. 5C′,C″). By contrast, overexpression of BAF155 did not affect the mobility of CENPA (*n*=9; 93.1±1.63% compared with 94±1.7% for the control, Fig. 5C′,C″), indicating a specific effect of BAF155 on BAF57. Together, these results support the hypothesis that the BAF complex is dynamic in early embryos and can be modulated by the level of BAF155.

**Fig. 6.**
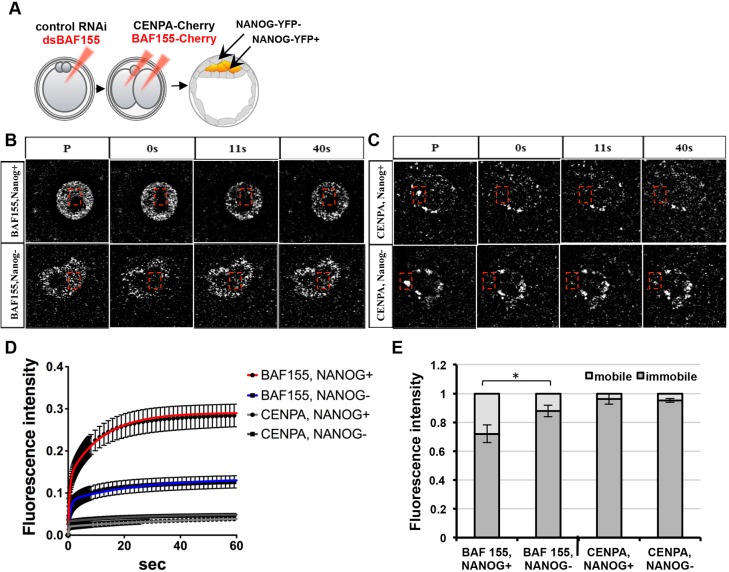
**NANOG^+^ cells contain more mobile BAF155 than NANOG^−^ cells.** (A) Rescue conditions in *Nanog-YFP* transgenic embryos were applied to measure the live kinetics of BAF155 between the lineages at E4.5. The kinetics of CENPA were measured in embryos injected with control RNAi at the zygote stage (*n*=11). (B,C) The recovery kinetics of BAF155 and CENPA were assessed in a rectangular region of nuclei after photobleaching. The fluorescence intensity was measured prior to photobleaching (P) and for 40 s during the recovery phase after photobleaching (0 s). (D) NANOG^+^ cells show greater recovery of BAF155 than NANOG^−^ cells, whereas recovery of CENPA is similar. (E) NANOG^−^ cells have a significantly higher immobile fraction of BAF155 protein (87.7±4.3%) than NANOG^+^ cells (72.06±6.2%); *P<0.05, *F*-test. The difference in the size of the immobile fraction of CENPA between NANOG^−^ (95.17±1.2%) and NANOG^+^ (96.31±3.6%) cells was not statistically significant (*P*>0.05, *F*-test). Error bars represent s.e.m.

As our findings indicated higher levels of BAF complex in the extra-embryonic versus embryonic lineages (Fig. 1C-F) we next used FRAP to assess the mobility of BAF155 in both lineages. To this end, we have established a transgenic line in which *Nanog* is fused to *YFP* by direct knock-in as a live marker of pluripotent cells (Fig. S6A-C, Movie 3). We introduced fluorescently tagged BAF155 into embryos with a downregulated level of BAF155 using previously established rescue conditions (Fig. 6A, Fig. 3I-K). FRAP measurements for BAF155 (*n*=16) and CENPA (*n*=11) revealed that whereas there was little difference in the mobility of CENPA between NANOG^+^ (EPI) and NANOG^−^ (TE) cells (96.31±3.6% and 95.17±1.2%, respectively, *P*>0.05, *F*-test), 87.79±4.03% of BAF155 was immobile in NANOG^−^ cells versus 72.06±6.2% in NANOG^+^ cells (*P*<0.05, *F*-test) (Fig. 6B-E). This increased amount of immobile BAF155 in NANOG^−^ cells is in accordance with the increased proximity of BAF155 to other core BAF complex proteins as measured by PLA in extra-embryonic lineages prior to implantation (Fig. 1A,B). These results suggest that greater levels of stable BAF complex in cells promotes commitment to the extra-embryonic lineage.

### CARM1-mediated methylation of BAF155 influences assembly of the BAF complex and lineage specification

**Fig. 7.**
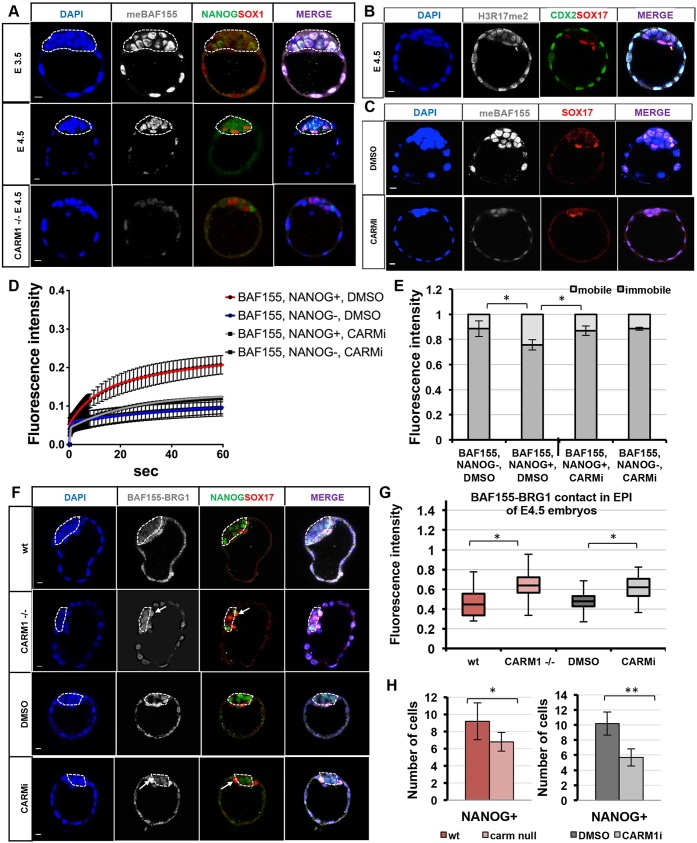
**CARM1-mediated methylation of BAF155 influences assembly of the BAF complex and lineage specification.** (A) Methylated BAF155 in E3.5 and E4.5 embryos. Methylated BAF155 was detectable at only low levels in *Carm1*^−/−^ embryos. (B) The distribution of H3R17me2 at E4.5. (C) Methylation of BAF155 is reduced in embryos treated with a CARM1-specific inhibitor (CARMi) and is unaffected by DMSO carrier. (D) NANOG^+^ cells in CARMi-treated embryos show reduced FRAP recovery of BAF155-mCherry compared with NANOG^+^ in DMSO-treated embryos. (E) NANOG^+^ cells (CARMi) have a higher immobile fraction of BAF155 protein (75±3.6%) than NANOG^+^ cells in DMSO (86.87±2%). (F) The frequency of interactions between BAF155 and BRG1 is comparably increased in EPI cells (dashed outline) of *Carm1* null and CARMi E4.5 embryos (arrows), in contrast to EPI cells of wild-type and DMSO-treated embryos. (G) The fluorescence intensity generated by BAF155-BRG1 association is increased in EPI cells of *Carm1*^−/−^ compared with wild-type embryos, and in CARMi-treated compared with DMSO-treated embryos. (H) The number of NANOG^+^ cells is decreased in *Carm1* null and CARMi embryos. Error bars represent s.e.m. *P<0.05, **P<0.001, Student's *t*-test. Scale bars: 10 μm.

BAF155 is reported to be one of the prime targets of CARM1 (Wang et al., 2014), an epigenetic modifier with a role in cell fate specification in the mouse embryo and ESCs (Torres-Padilla et al., 2007; Parfitt and Zernicka-Goetz, 2010; Wu et al., 2009), which led us to ask whether methylation of BAF155 by CARM1 contributes to the difference in its mobility in pluripotent versus differentiating cells in the embryo. Examining the localisation of the methylated form of BAF155 (Wang et al., 2014) revealed that meBAF155 is distributed equally between the lineages at E3.5 (*n*=8, Fig. 7A, Fig. S7A, *P*>0.05, ANOVA), whereas at E4.5 it is present at significantly lower levels in TE than in other lineages (*n*=6, Fig. 7A, Fig. S7B, *P*<0.001, ANOVA). In agreement, we found that only low levels of meBAF155 were detectable in *Carm1* knockout embryos (Kim et al., 2010) (Fig. 7A, *n*=5), in line with arginine methylation of BAF155 being CARM1 dependent (Wang et al., 2014). To address whether a similar TE-specific decrease is present for other CARM1-mediated methylations, we also compared the levels of H3R17me2 among the three distinct lineages of E4.5 blastocyst (Fig. 7B, Fig. S7C). We discovered that there are no significant differences in H3R17me2 levels (Fig. S7C), suggesting that the difference in the methyltransferase activity of CARM1 between the lineages is unlikely to be a consequence of decreased meBAF155, but rather of the difference in the availability of BAF155 as a substrate for CARM1 methylation in TE.

To further assess the effect of CARM1 on BAF155 methylation, we used a specific CARM1 inhibitor (CARMi) (Cheng et al., 2011). The addition of CARMi at the zygote stage resulted in developmental arrest after the first division (Fig. S7D, *n*=18), whereas its addition at the 2-cell stage had a range of developmental effects depending on the concentration of the inhibitor (Fig. S7E). We selected an intermediate concentration (9 µM) that led to substantial reduction in BAF155 methylation (Fig. 7C) while allowing 73% (*n*=22) of embryos to develop to the blastocyst stage [compared with 94.2% (*n*=34) in DMSO, Fig. S7E]. To determine the mobility of BAF155 upon CARMi treatment, we performed FRAP using rescue conditions (Fig. 3I-K). Whereas control embryos exhibited higher mobility of BAF155 in NANOG^+^ compared with NANOG^−^ cells (Fig. 7D,E, *P*<0.05, *F*-test), consistent with our previous observations (Fig. 6D,E), the mobility of BAF155 in NANOG^+^ cells of CARMi-treated embryos was decreased compared with NANOG^+^ DMSO-treated control embryos (Fig. 7D,E, *P*<0.05, *F*-test). This suggests that methylation of BAF155 by CARM1 can influence the mobility of BAF155 in pluripotent NANOG-expressing EPI cells.

To test whether the reduction in CARM1-mediated methylation affects the associations of the BAF complex subunits, we analysed the proximity of BAF155 and BRG1 in E4.5 *Carm1*^−/−^, CARMi-treated and control (DMSO-treated) embryos by PLA. Consistent with our previous observations (Fig. 1C,D), BAF155-BRG1 proximity was decreased in the EPI of control (*n*=9) and wild-type (*n*=6) embryos (Fig. 7F,G). By contrast, the signal from BAF155-BRG1 proximity was increased in the EPI and in some extra-embryonic cells of *Carm1*^−/−^ (*n*=8) and CARMi-treated (*n*=5) embryos compared with control groups (Fig. 7F,G, *P*<0.05, ANOVA). Together, the FRAP and PLA analyses suggest that levels of stable BAF complex are increased in the absence of CARM1 function.

Finally, to test whether the increased proximity of BAF155 with BRG1 in EPI upon CARM1 inhibition would affect lineage specification, we examined the number of NANOG^+^ cells in *Carm1* knockout (*n*=8) and CARMi-treated (*n*=21) embryos. This revealed a significant reduction in the number of NANOG^+^ cells compared with control embryos (Fig. 7H).

Together, these results suggest an important role of CARM1-mediated methylation of BAF155 in normal development: its absence leads to increased proximity of BAF155 with BRG1, stabilisation of the complex and a decrease in the number of NANOG^+^ pluripotent cells.

## DISCUSSION

Epigenetic changes to chromatin play a profound role in mouse development. Elucidating the mechanisms that control these changes is, however, challenging at present due to technical difficulties associated with studying protein interactions on a single-cell level in mouse embryos in which classical biochemical approaches are not possible. To overcome these technical restrictions we have applied PLA in combination with FRAP experiments to gain insight into how the composition of the core BAF complex is established in different lineages. We found that a major component of the BAF chromatin remodelling complex, BAF155, plays a crucial role in regulating the dynamics of the BAF complex in the early mouse embryo, and is essential for cell fate specification before the implantation stage.

We demonstrate that, prior to implantation, the proximity of the core BAF complex subunits BAF155, BRG1 and BAF57 increases in the extra-embryonic lineages. We then find a reduced mobility of BAF155 in the extra-embryonic compared with the embryonic lineages, which suggests either increased complex formation or stability. We further demonstrate that a decreased level of CARM1-mediated BAF155 methylation is at the heart of the increased BAF complex stability in the extra-embryonic lineages. The functional importance of BAF155 in development is indicated by the opposing responses to its expression levels: reduced levels BAF155 lead to increased expression of the pluripotency marker *Nanog*, whereas upregulated BAF155 increases the expression of differentiation marker genes.

These findings have several important implications. First, they suggest a regulatory function of chromatin remodelling by the BAF complex during early mouse development, rather than a permissive role. Second, the differential BAF complex subunit associations between the lineages and the consequences of their misexpression imply that the BAF complex is involved in the establishment of pluripotency and extra-embryonic transcriptional programmes. Finally, CARM1-mediated regulation of BAF complex dynamics, alongside its known role in histone tail modification (Torres-Padilla et al., 2007), emphasizes the interconnectivity of epigenetic mechanisms required to ensure correct cell fate programmes and prepare an embryo for implantation. Cumulatively, these results indicate the role of the BAF complex in lineage specification of the mouse embryo, suggesting that the developmental arrest of embryos lacking the subunits of the BAF complex at peri-implantation stages (Bultman et al., 2000; Guidi et al., 2001; Kim et al., 2001; Klochendler-Yeivin et al., 2000) could be due to a failure to accurately specify extra-embryonic from pluripotent cell fates.

The involvement of the BAF complex in pluripotency has previously been studied in ESCs. Proteomic studies revealed that the BAF complex in ESCs (esBAF) has a distinctive composition defined by the presence of pluripotency-specific subunits (Ho et al., 2009b). Furthermore, it was shown that esBAF occupies the enhancers and promoters of many genes of the pluripotency network, including *Oct4*, *Nanog* and *Sox2*, as well as their targets, suggesting a functional interaction between esBAF and the pluripotency network (Ho et al., 2009a). Knockdown of BRG1 was demonstrated to have a dual effect in ESCs: acute depletion resulted in immediate upregulation of *Nanog* and *Oct4* (Singhal et al., 2014), whereas depletion using a conditional allele and shRNA led to initially maintained expression of these genes but their downregulation after several days (Kidder et al., 2009; Ho et al., 2009b). It has been suggested that the role of BRG1 in the pluripotent cells is to tonically repress the expression of *Nanog* and *Oct4*, so as to maintain the pluripotency network (Ho et al., 2009b). Similarly, knockdown of BRG1 in a blastocyst has been shown to derepress *Nanog* and *Oct4* expression, suggesting that BRG1 is also a negative regulator of these genes in the early embryo (Kidder et al., 2009; Carey et al., 2015). Much less is known about the role of other components of the BAF complex in pluripotency. One report demonstrating that, upon robustly triggered differentiation in ESCs, BAF155 instigates changes in chromatin to repress *Nanog*, has resonance with our findings (Schaniel et al., 2009).

The surprising time-dependent effect of downregulation of the core BAF complex subunits could suggest a dynamic requirement for the BAF complex in the regulation of pluripotency. This could be controlled through changes in BAF complex stoichiometry or through mobilisation dynamics of existing subunits. The findings we present here suggest that differences in the level of mobilised BAF155 between pluripotent and extra-embryonic lineages is key for the differential cell type-specific regulatory effect of the BAF complex on *Nanog* and other lineage specification genes. How might this difference in BAF155 mobilisation between the lineages be controlled? One possibility is that it is influenced by post-translational modifications of its subunits. A recent study (Wang et al., 2014) reported that BAF155 is modified by methylation specifically at R1064. Although this methylation does not drastically affect incorporation of BAF155 into the BAF complex, meBAF155 does not form a complex with the catalytic subunit BRG1 and others at specific transcriptional sites (Wang et al., 2014). The methyltransferase found to modify BAF155 is CARM1, which is implicated in cell fate decisions during early embryogenesis (Parfitt and Zernicka-Goetz, 2010; Torres-Padilla et al., 2007).

**Fig. 8.**
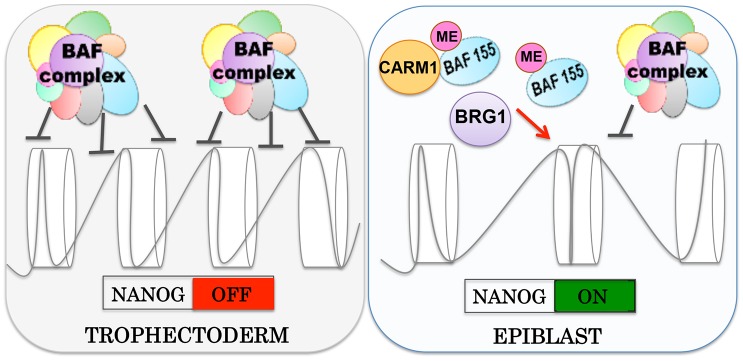
**Model for CARM1-mediated regulation of** ***Nanog*** **expression by the BAF complex.** Prior to implantation, expression of the pluripotency gene *Nanog* needs to be repressed in TE and tightly controlled in EPI. BAF complexes in TE, which have an increased stability of BAF155-BRG1 contact, act to repress the expression of *Nanog*. In EPI, however, the stability of the BAF complex is decreased through CARM1-mediated methylation of subunit BAF155. This mechanism modulates the expression of *Nanog* that is compatible with further embryonic development.

We found that, just prior to implantation, methylated BAF155 is decreased in TE in comparison to EPI. CARM1 inhibition triggered low BAF155 methylation, elevated BAF155-BRG1 association and a decreased number of *Nanog*-positive cells. This has resonance with the previous findings that CARM1-triggered methylation of histone H3 arginine residues is required to promote pluripotency (Torres-Padilla et al., 2007). We suggest that refining the levels of expression of *Nanog* in EPI could occur through regulation of BAF complex assembly dynamics via CARM1-triggered methylation of its core subunit BAF155. This methylation limits the number of BAF155-BRG1-containing complexes formed, and therefore alleviates the repressive effect they have on the expression of *Nanog*. This regulatory mechanism of the assembly dynamics of the BAF complex could contribute to the modulation of expression levels of pluripotent markers such as *Nanog* in EPI, controlling the level of expression so that it is compatible with further development. Conversely, the absence of such a pathway in TE results in a low level of BAF155 methylation, high BAF155-BRG1 proximity and is correlated with the silencing of *Nanog* that earmarks the cells for extra-embryonic differentiation (Fig. 8).

The question of how repression of *Nanog* in TE is achieved mechanistically by the BAF155-BRG1-containing complex remains open. It was recently suggested to occur through the interaction of BRG1 with HDAC1 specifically in TE, which antagonises histone acetylation at the proximal enhancer of *Nanog* and thereby shuts down its expression (Carey et al., 2015).

In summary, our results demonstrate the significance of interconnected epigenetic regulation in the specification and maintenance of cell fates in the early mouse embryo. Although we know several of the key players that are involved in the establishment of pluripotent and extra-embryonic fates, there is still little information about how these mechanisms are coordinated *in vivo*. The challenge for the future will be to determine the precise molecular mechanisms that direct the differences in epigenetic programming in individual cells as the embryo progresses through its normal development.

## MATERIALS AND METHODS

### Embryo collection and culture

6- to 8-week-old F1 females from C57B16×CBA crosses were superovulated by injection of 10 IU PMSG (Intervet) and 10 IU human chorionic gonadotropin (hCG; Intervet) 48 h later and mated with F1 males expressing *CAG-GFP*, *Nanog-YFP* or that were *Carm1*^−/−^ (Kim et al., 2010). Oviducts were dissected in M2 medium with bovine serum albumin (BSA) and cultured in KSOM as previously described (Bischoff et al., 2008). The selective CARM1 inhibitor *bis*-benzylidene piperidinone (Millipore) was dissolved in dimethyl sulfoxide (DMSO). Culture of ESCs and TSCs is described in the supplementary Materials and Methods. Single-embryo genotyping by PCR and the primers used are described in the supplementary Materials and Methods and Table S5.

### Microinjections

mRNAs for microinjection were produced by *in vitro* transcription of *Sfi*I-linearised RN3P or *Hpa*I-linearised pCS2+ plasmids using mMessage mMachine T3 or SP6 RNA polymerase (Life Technologies) according to the manufacturer's instructions. CENPA plasmid was a gift from D. Glover, University of Cambridge, UK; BAF155 and Baf57 were gifts from G. Crabtree lab, University of Stanford, CA, USA. The generation and sequences of dsRNAs are listed in the supplementary Materials and Methods and Table S1. Microinjection of mRNAs or dsRNAs was performed as described (Zernicka-Goetz et al., 1997).

### Image acquisition and analysis

Live time-lapse images were collected every 15 min on an inverted Zeiss Axiovert spinning disk confocal system (Intelligent Imaging Solutions) using a 63×/1.3 NA water objective. Image acquisition from fixed preparations was carried out using a Leica SP5 confocal microscope with a 40×/1.4 NA oil-immersion objective. Analysis of images and creation of image *z*-projections were performed in Fiji (Schneider et al., 2012). Automatic quantification was performed using the Object Scan plugin for Fiji (see supplementary Materials and Methods).

### Immunofluorescence and PLA

Protocols for immunofluorescence on fixed embryos and cells, including a list of the antibodies used, and an extended PLA protocol adapted for mouse embryos are presented in the supplementary Materials and Methods and Table S2.

### cDNA constructs

Human *BAF155* (NM_003074.3), human *BRG1* (NM_001128844.1), mouse *Baf57* (NM_020618.4) and human *CENPA* (gi602413) were subcloned into RN3P and pCS2+ for the *in vitro* transcription of mRNA.

### RNA isolation and quantitative PCR

Transcripts were isolated using the Arctus PicoPure RNA Isolation Kit (Applied Biosystems). qRT-PCR reactions were performed in triplicate using the Power SYBR Green PCR RNA-to-CT 1-Step Kit (Applied Biosystems) and the primers listed in Table S3.

### Generation of *Nanog-YFP* reporter mice

The C-terminus of NANOG was tagged with YFP protein as described previously (Skarnes et al., 2011; Hofemeister et al., 2011). Details of the generation of the line are provided in the supplementary Materials and Methods and Table S4.

### FRAP data analysis

Recovery curves were obtained by measuring the intensities of 18-μm^2^ background, control and photobleached regions using Leica FRAP Wizard software. FRAP data acquisition and the parameters and equations used for the analysis are listed in the supplementary Materials and Methods and Table S6. GraphPad Prism software was used for nonlinear fitting and plotting of graphs.

### Statistical analysis

ANOVA was used to test statistical significance when comparing means of more than two independent groups. Student's *t*-test was used to compare the means of two independent groups. *F*-test was used for FRAP data analysis in order to identify the model that best fits the population from which the recovery data were sampled.
